# Agricultural Publications

**Published:** 1872-12

**Authors:** 


### AGRICULTURAL PUBLICATIONS.

Away out yonder in the valley of the Mississippi, and nigh upon its banks, in the very midst of the blue grass region of old Kentuck, are two men, on the sunny side of forty, conducting an agricultural paper, with an elevation and a refinement and a culture in all the articles, editorial, original, and selected, far better for Sunday reading than some of our "religious" newspapers. Both these gentlemen would most decidedly object to having anything written for public eyes, bearing on the privacies of life; but these are introduced here for satisfactory reasons of a practical character. They are both men of a thorough collegiate education, one of them a graduate of Harvard; both born in the West, both young, both married early, and have half a dozen or more children each; both strictly trained from earliest infancy by mothers who were Presbyterians by inheritance from remote generations; both free from the prevalent vices of youth, drinking, gaming, and tobacco; both have had military appointments; both born to large wealth, knowing how to keep and increase it, and are brothers-in-law.

In this narration there are two striking contradictions of two very generally received statements. First, that rich men's sons are pretty sure to grow up worthless. Second, that strict, religious training, or being kept in the house on Sundays, made to go to church as a matter of course, and compelled to learn the catechism, has naturally the effect to implant such a dislike against religion, as to end in an indignant escape from all these tyrannies, the moment of passing beyond parental control.

This diversion was not intended when the heading of the article was written; but there is a moral in the story, of a practical character, which every reader will do well to make a note of. It is not known that either of the editors are members of any church; but it is a fact that they both have always had sturdy good health; and that their health and fortunes and mental culture and high social position are all brought into requisition, in the amateur employment of editing a weekly newspaper, at two dollars a year, to reach farmers' families, well calculated to diffuse useful knowledge, and to promote good morals and domestic virtue; contrasting strongly with the manner in which many newspapers are conducted in the East, and magazines also, whose columns are so frequently blurred by ill-bred flippancies in reference to the Christian religion, its ministers, its friends, its Bible, and its Sabbath day, to say nothing of inane and vile love stories, with disgusting narrations of divorce trials and rapes and murders and adulteries, with pictures to match, indecent, filthy, and vile.

It is creditable to the agricultural press, all over the nation, that they are invariably edited in a manner becoming their mission, like the "Farmer's Home Journal," Lexington, Ky. The pages of this journal abound with a miscellaneous reading, well fitted to be spread on the tables of farmers' families, and to meet the eyes of sons and daughters, with nothing to offend the most refined taste, encouraging all that is good, and reprobating evil doing everywhere.

